# Cellular Immuno-Profile in Septic Human Host: A Scoping Review

**DOI:** 10.3390/biology11111626

**Published:** 2022-11-07

**Authors:** Christian Zanza, Giorgia Caputo, Gilda Tornatore, Tatsiana Romenskaya, Andrea Piccioni, Francesco Franceschi, Marco Artico, Samanta Taurone, Gabriele Savioli, Yaroslava Longhitano

**Affiliations:** 1 Foundation “Ospedale Alba e Bra”, Department of Emergency Medicine, Anesthesia and Critical Care Medicine, Michele and Pietro Ferrero Hospital, 12060 Verduno, Italy; 2 Department of Emergency Medicine, Policlinico Gemelli-RCCS-Catholic University of Sacred Heart, 00168 Rome, Italy; 3 Department of Anesthesia and Critical Care Medicine, St. Antonio and Biagio Hospital, 15121 Alessandria, Italy; 4 Department of Anesthesia and Intensive Care Medicine, University of Milan-Bicocca, 20126 Milan, Italy; 5 Department of Sensory Organs, Policlinico Umberto I, Sapienza University of Rome, 00185 Rome, Italy; 6 Department of Movement, Human and Health Sciences—Division of Health Sciences, University of Rome “Foro Italico”, 00135 Rome, Italy; 7 Department of Emergency Medicine, Polyclinic IRCCS S. Matteo, University of Pavia, 27100 Pavia, Italy

**Keywords:** blood purification, flow cytometry, immune cells, inflammation, lymphocytes, monocytes, neutrophils, sepsis, COVID-19

## Abstract

**Simple Summary:**

Septic shock is a life-threatening disease caused by a dysregulated host response to infection, affecting millions of people every year and killing more than 25% directly despite advances in modern medicine. This pathology is characterized by apoptosis-induced depletion of immune cells and immunodepression. Many alterations in the expression of surface markers of neutrophils and monocytes have been described in septic patients. There is no specific treatment but the early identification and diagnosis of the pathology as well as timely treatment can greatly improve patient outcomes. The aim of this study was to inspect the recently published literature to inform the clinician about the most up-to-date techniques for the study of immune cell phenotypes and on the function of leukocytes of extracorporeal and non-blood purification treatments proposed for sepsis were also analyzed. The most important alteration observed in septic neutrophils is the activation of a survival program capable of resisting apoptotic death. As regards adaptive immunity, sepsis-induced apoptosis leads to lymphopenia in patients with septic shock and this process involves all types of T cells (CD4, CD8 and Natural Killer), except for regulatory T cells, favoring immunosuppression. Several promising therapies that target the host’s immune response to sepsis are currently under evaluation.

**Abstract:**

Innate and adaptive immune system cells play a critical role in the host response to sepsis. Sepsis is a life-threatening disease characterized by apoptosis-induced depletion of immune cells and immunodepression, which contribute to morbidity and mortality. Many alterations in the expression of surface markers of neutrophils and monocytes have been described in septic patients. The aim of this study was to inspect the recently published literature to inform the clinician about the most up-to-date techniques for the study of circulating leukocytes. The impact on cell phenotypes and on the function of leukocytes of extracorporeal and non-blood purification treatments proposed for sepsis were also analyzed. We conducted a systematic review using Pubmed/Medline, Ovid/Willey, the Cochrane Library, the Cochrane Controlled Trials Register, and EMBASE, combining key terms related to immunological function in sepsis and selected the most relevant clinical trials and review articles (excluding case reports) published in the last 50 years. The most important alteration in neutrophils during sepsis is that they activate an anti-apoptotic survival program. In septic monocytes, a reduced characteristic expression of HLA-DR is observed, but their role does not seem to be significantly altered in sepsis. As regards adaptive immunity, sepsis leads to lymphopenia and immunosuppression in patients with septic shock; this process involves all types of T cells (CD4, CD8 and Natural Killer), except for regulatory T cells, which retain their function. Several promising therapies that target the host immune response are currently under evaluation. During the worldwide pandemic caused by SARS-CoV-2, it was useful to study the “cytokine storm” to find additional treatments, such as the oXiris^®^ filter. This therapy can decrease the concentration of inflammatory markers that affect the severity of the disease.

## 1. Introduction

Sepsis is an important public health issue globally. Septic shock is a life-threatening disease caused by a dysregulated host response to infection, affecting millions of people every year and killing more than 25% directly despite advances in modern medicine. Maybe this reflects the increasing age of the population with more comorbidities [1,2,3].

Given the large percentage of elderly patients worldwide, it is likely that sepsis will become an even greater problem in the future.

A consensus conference in 1991 defined “sepsis” as the combination of an infection with two or more features of the “systemic inflammatory response syndrome” (SIRS): a high or low body temperature, an elevated respiratory and pulse rate and anomalies in the white blood cell count.

According to the Third International Consensus Definition for Sepsis and Septic Shock (Sepsis-3), the criteria are expanded, and sepsis is now defined as a life-threatening organ dysfunction characterized by a complex series of cellular changes in response to an infection or other dangerous signs [4,5,6] (Table 1 and Table 2). This was very important because it shifted attention to the immune system reaction for the first time, rather than remaining on the pathogen responsible for the infection.

We do not fully understand the pathogenesis of sepsis and there is no specific treatment but it is clear that the early identification and diagnosis of the pathology as well as timely treatment can greatly improve patient outcomes.

The cellular changes during sepsis are triggered by some receptor patterns—Toll-like receptors (TLR), NOD-like receptors (NLRs), RIG-I helicases, and C-type lectin receptors, expressed on most types of cells [7]. These receptors are activated through the expression or inhibition of many immune and metabolic genes, and through post-translational changes in the main intracellular proteins involved in signaling and transcriptional regulation.

The cells of the innate and adaptive immune system are important in the host’s response to infection, and so in sepsis. Furthermore, leukocytes may be a good parameter for the evaluation of the altered immune response in sepsis, because they are involved in the response to acute injury and they are quite easy to sample in peripheral blood.

## 2. Materials and Methods

We conducted a systematic review using Pubmed/Medline, Ovid/Willey, the Cochrane Library, the Cochrane Controlled Trials Register, and EMBASE, combining key terms related to immunological function in sepsis and selected the most relevant clinical trials and review articles (excluding case reports) published in the last 50 years. 

After that research, we have focused on 5 key immunological issues that physicians need to consider when assessing sepsis:

1. Characteristic changes in neutrophil and monocyte function in sepsis.
2. Characteristic functional and phenotypic changes in adaptive immune system cells during sepsis.
3. Techniques that are useful for the study of circulating cells in sepsis and to understand if immune cells act as a “biopsy sample”.
4. Can extracorporeal and non-blood purification therapies alter cell phenotypes and/or change the function of leukocytes?
5. COVID-19 and a “cytokine storm”: the role of blood purification.

To address these issues, we searched for evidence using the Cochrane Controlled Trials Register, the Cochrane Library, Medline, Embase, and Scopus from 1966 to more recently. Finally, we reviewed the results with the group and used the Delphi method to obtain unanimous consensus.

## 3. Discussion

### 3.1. Characteristic Changes in Neutrophil and Monocyte Function in Sepsis

Polymorphonuclear neutrophils (PMNs) and monocytes are predominant circulating phagocytic cells of the innate immune system; they are derived from common bone marrow precursors and their biological destiny differs in the bloodstream [8,9], where they play a fundamental role in regulating innate and adaptive immunity.

Some studies show a reduction in monocyte count that seems to be correlated with the severity of sepsis, the risk of mortality, the rate of bacteriemia and organ dysfunction. Hyunwoo Chung et al. enrolled 2012 patients with severe sepsis and showed that the neutrophil counts were significantly increased and the lymphocyte counts were significantly decreased in both survivors and non-survivors (*p* < 0.01, respectively). On the other hand, the monocyte counts were significantly increased in survivors and decreased in non-survivors (*p* < 0.01, respectively). In this latter group of patients, there were significantly higher rates of bacteremia, mechanical ventilation, and crude 28-day mortality (*p* < 0.001). This was probably due to low monocyte counts corresponding to poor local infection control and spreading to systemic evolution [10].

On damage, neutrophils can extrude their DNA to create extracellular neutrophilic traps (NETs), which serve to trap bacteria and activate local coagulation mechanisms [11].

Ahmed and colleagues studied neutrophil migration and behavior at the inflammation site on skin samples. Neutrophils had less capacity to migrate from peripheral blood, but the phagocytic, bactericidal capacities increased and oxidative capacity was unmodified if compared to healthy controls [12]. Others have shown that while basal neutrophil activation is enhanced in sepsis, the ability to respond to a de novo stimulus is reduced [13].

Among the most important changes observed in septic neutrophils is their ability to activate a survival program that counteracts the apoptotic pathway they encounter after leaving the bone marrow [14]. While 50% of resting neutrophils will show the typical morphological changes in the apoptosis process after 24 h of in vitro culture, the corresponding rate for septic neutrophils is only 5–10% [15].

**Figure 1:**
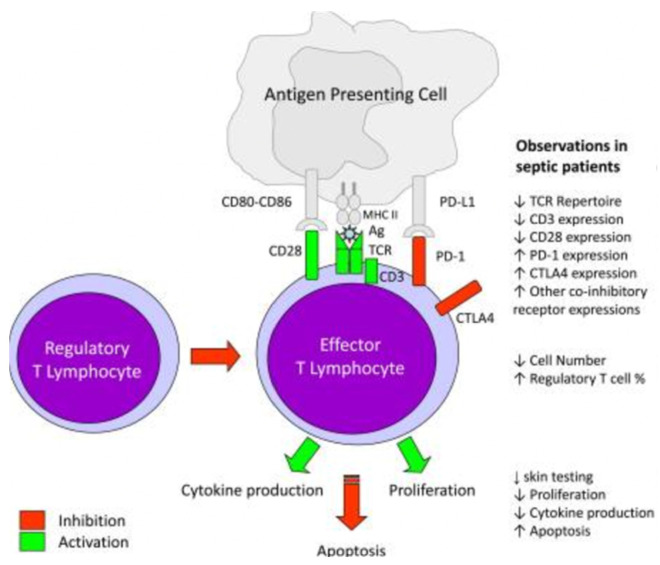
T lymphocyte alteration in sepsis.

De novo gene expression is necessary to prolong neutrophil survival and interleukin-1b is necessary for this process [16] in addition to PBEF/Nampt—a protein that represents a break for the biosynthesis rescue pathway of NAD [17]. The reduced expression of the main HLA-DR histocompatibility antigen is a characteristic finding on septic monocytes [18]. This is related to an increased risk of complications from infection and death [19], so it has been suggested to use HLA-DR levels as a potential biomarker to estimate the success of the therapies used for sepsis [20,21] (Figure 1).

Evidence suggests that immature neutrophils in the circulatory stream during sepsis may undergo a differentiation process in monocytic cells [22], underlining the plasticity of the response of myeloid cells to an acute insult and in sepsis. Two alterations in the cellular phenotype deserve special mention.

**Figure 2:**
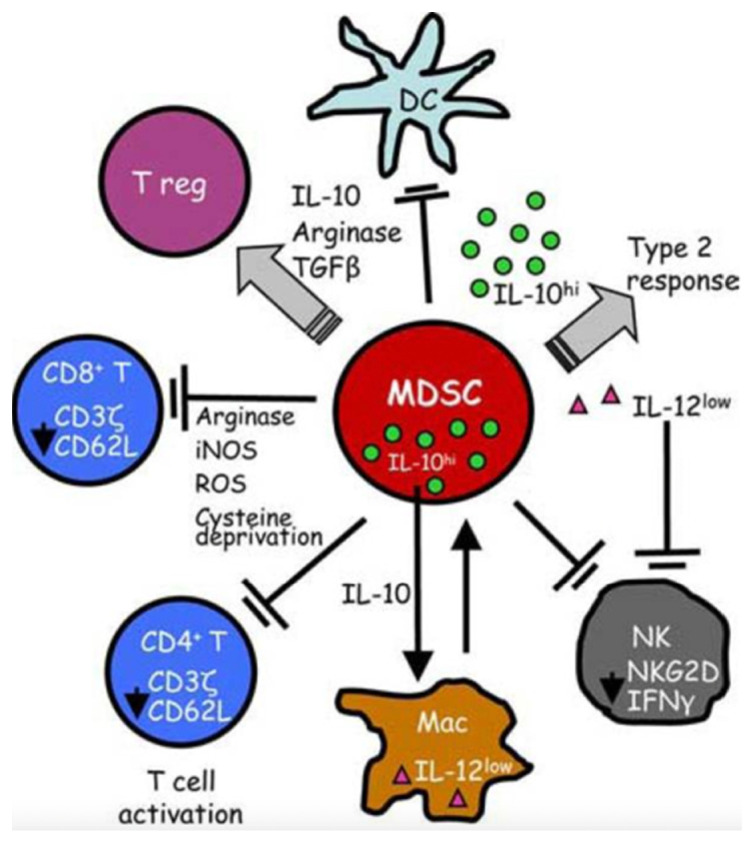
MDSCs suppress antitumor immunity through a variety of mechanisms. T cell activation is suppressed by the production of arginase and ROS, cysteine deprivation and the induction of Tregs. Innate immunity is impaired by the down-regulation of macrophage-produced IL12, the production of IL10 and the suppression of NK cells.

**Figure 3:**
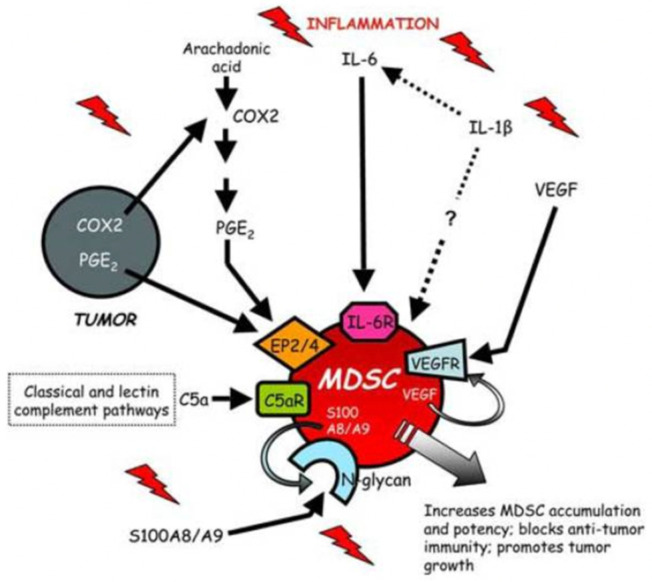
MDSCs are induced and/or activated by multiple proinflammatory mediators. MDSCs accumulate in the blood, bone marrow, lymph nodes and at tumor sites in response to proinflammatory molecules produced by tumor cells or by host cells in the tumor microenvironment.

Myeloid-derived suppressor cells (MDSCs) circulate as CD34^+^ and CD11b^+^ and can inhibit the adaptive immune response, in particular the activation of T cells [23,24]. Their ability is to generate reactive oxygen species (ROS) and arginase products. MDSCs are protective in animal sepsis models [25,26]: instead, their role in the human body during sepsis is unclear; their presence is associated with lymphopenia and increased mortality [27] (Figure 2 and Figure 3).

As mentioned, PMNs are modified in number or function [28]; in several animal models, they are involved in non-selective tissue damage [29], releasing ROS and proteases such as elastase [30].

To confirm what has been said, sepsis in the human body is associated with delayed neutrophil apoptosis, as mentioned from Taneja et al. [31,32].

Furthermore, neutrophils extracted from the blood of patients who have suffered from sepsis or polytrauma can induce the apoptotic death of other cells by the dephosphorylation of Caspase-8 on the epithelial cell [33]. The expression of PDL-1 is increased on septic neutrophils and, through interaction with PD-1 lymphocytes, it can induce the apoptotic death of CD4+ lymphocytes [34].

### 3.2. Characteristic Functional and Phenotypic Changes in Adaptive Immune System Cells during Sepsis

Functional and phenotypic changes in the adaptive immune system in sepsis can be summarized as follows: the cooperation between innate and adaptive immune systems; the mechanism and the phenotypic changes observed over time.

**Figure 4:**
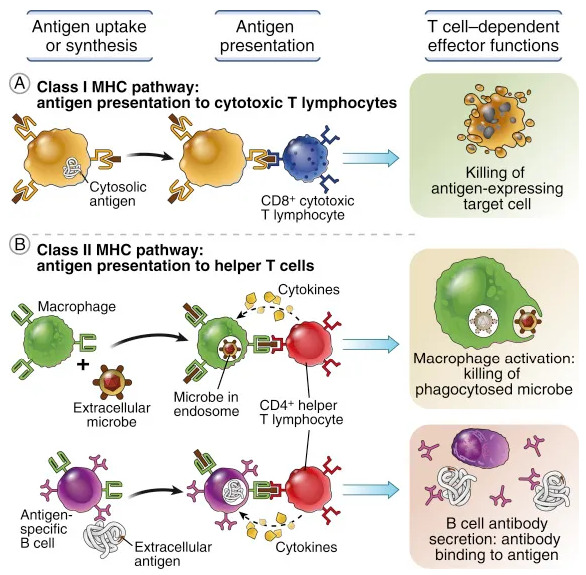
Antigen capture and presentation to lymphocytes.

The adaptive immune system is composed of T and B lymphocytes and uses antigenic receptors to solve their function. This is possible thanks to the recognition by the innate immune system of microbial patterns (PRRs). An important role is represented by “antigen-presenting cells” (APCs). After processing microbial antigens, APCs present the antigen to native T cells via major histocompatibility complex (MHC) molecules. In addition, activation of T cells requires a series of stimulating signals and an environment rich in cytokines. The activation of B cell receptors, which can take place through different pathways, leads to the production of specific antibodies in the organism (Figure 4).

Then, whatever the pathogen detected, the direct activation of the dendritic cells (DCs) by the PRR activates the response of the T cells. The organization of the immune response changes over time after the initial insult and follows different scenarios. The immediate response is identified by “an inflammatory storm”, which sees the release of both pro- and anti-inflammatory factors, activation and cellular cooperation in order to eradicate the infection.

The immune system can sometimes exceed this response, and this can lead to organ failure, which is responsible for the early death in at least 50% of septic shock cases [35,36].

**Figure 5:**
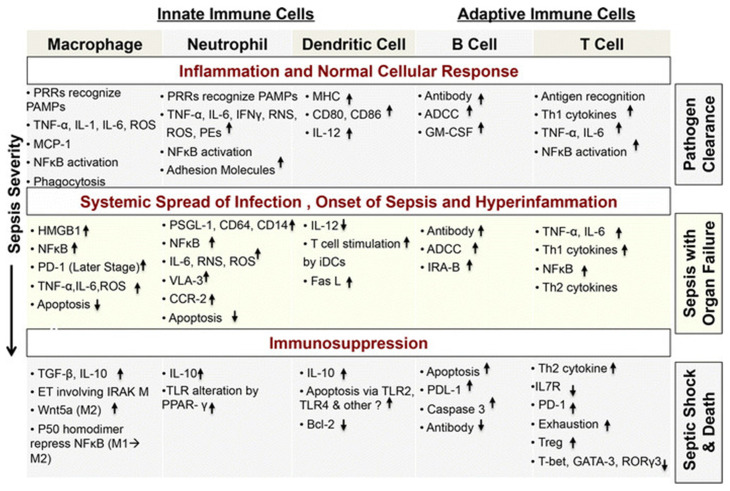
Overview of cellular changes occurring during sepsis. The first line shows the correct activation of the immune system during infection. If the innate and adaptive immune systems fail to contain the ongoing infection locally, the infection spreads systemically, triggering a hyperinflammatory innate and adaptive immune response. Further progression of infection and spread of the dysfunctional and altered cellular responses, including changed surface receptor expression, inappropriate inflammatory mediator secretion, and untimely apoptosis of immune cells, lead to the development of sepsis (middle panel). To regulate hyperinflammatory immune cell activities, the body goes through a loss of balance between inflammatory and anti-inflammatory response and a generalized immunosuppressive stage (bottom panel). As described in the figure, different phenotypic and molecular changes take place in immune cells as sepsis progresses. Thus, a host response that is designed to protect against pathogens causes tissue-damaging events, leading to multi-organ system failure and death. (Legend: ↑:increase; ↓: decrease).

**Figure 6:**
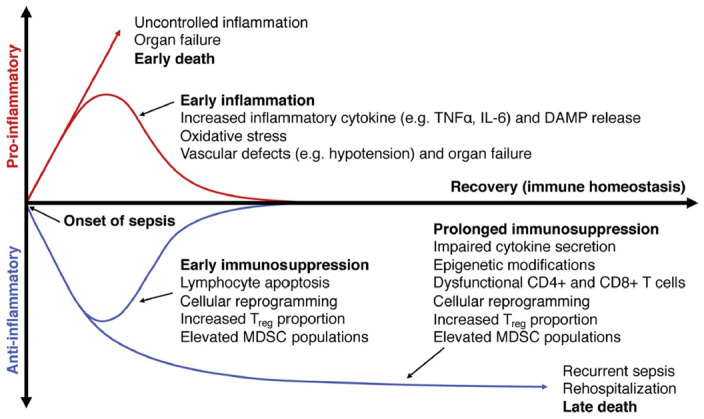
Stages of sepsis. Early in sepsis, both inflammation and immunosuppression occur concurrently. If inflammation is uncontrolled, this leads to organ failure and death. Those that avoid early death will either return to immune homeostasis, or progress to prolonged immunosuppression that continues after discharge. Prolonged immunosuppression predisposes survivors to infections, rehospitalizations, and ultimately to death. This phenomenon is marked by impaired cytokine secretion, dysfunctional T cells, and cellular reprogramming. Expansion of regulatory T cell and myeloid-derived suppressor cell (MDSC) populations also occurs early in sepsis and persists after sepsis, suggesting their role in maintaining this immunosuppressive phenotype.

Adaptive immunity changes that occur during this early phase (i.e., 5–7 days) have been studied less but remain a great challenge in the research for an innovative therapy [37] (Figure 5 and Figure 6).

The pioneering work of Zahorec et al. showed that surgical stress as well as systemic inflammation and sepsis determine important changes in the white blood cell count, characterized by neutrophilia and lymphopenia in correlation with the severity of the clinical course [38]. The neutrophil/lymphocyte ratio was used as a “stressor” to predict severity and/or outcome.

In 2002, Tschaikowsky et al. demonstrated that marked lymphopenia at sepsis onset was more pronounced in survivors on days 2, 3, 5 and 7 than in non-survivors [39]. Compared to healthy subjects, the reduction was 50% for CD4^+^ and CD8^+^ T cells in non-survivors and 75% in survivors. On days 1, 2 and 5, the percentage of both T cell subpopulations was approximately 2 fold that in non-survivors compared to survivors with a CD4/CD8 T cell ratio on days 1 and 2 significantly higher than the normal value (1.95 + 0.21). This ratio returned to normal on day 14, with no difference between survivors and non-survivors.

In the literature, there are few articles describing changes in lymphocyte subpopulations during sepsis. In 32 septic patients with purulent meningitis [40], a decrease in the absolute number of total T lymphocytes at hospitalization and rapid recovery after 7 days were observed. This lymphopenia involved CD4^+^, CD8^+^ and NK cells and was more pronounced with a Gram-positive infection. Compared to healthy volunteers, the reduced number of circulating B lymphocytes correlated well with the incidence of nosocomial infection.

The role of B cells in both innate and adaptive immune responses has become more important recently, especially after a mice study conducted by Scumpia ed al. in 2011, where an attenuated inflammatory response was shown to be linked to B cell deficiency [41].

Monserrat et al. enrolled 52 patients with septic shock and demonstrated that B cell activation and regulation markers at the time of admission seemed to be associated with a better outcome, with markers of apoptosis significantly lower in survivors than in non-survivors [42].

Several theories have been presented regarding the immune profile: elderly patients with numerous comorbidities can develop a limited initial hyperinflammatory phase, followed by an immunosuppression pattern; patients with early hyperinflammation sometimes experience immunosuppression until healing; others develop a state of prolonged immunosuppression that exposes them to secondary infections or to a recurrence of the initial unresolved infection [43].

Although the mechanisms behind these differences have not been fully identified, post-aggressive immunodepression (PAID), particularly for adaptive immunity, has been described several times in many inflammatory scenarios. The evolution towards PAID seems to be more frequent in septic patients [18].

Various threads of clinical evidence fit well with this theory: patients with sepsis and trauma lost the delayed hypersensitive response, a finding correlated with a higher mortality rate [44,45]; in sepsis, the reactivation of latent viruses such as cytomegalovirus and herpes simplex virus or secondary infections caused by relatively poorly virulent pathogens may also develop [18,46]. Blood tests during the late phase of sepsis show increased regulatory T cell counts (immunosuppressants) and an increase in the production of PD-1 and its L1 ligand [47,48]. The absolute number of all T cell types was reduced in septic or septic shock patients, except for regulatory T cells (circulating CD4^+^, CD25^+^, and Treg cells) [49]. Adaptive immunity cells up-regulate the expression of selected inhibitor receptors such as PD-1, with an expansion in the number of T suppressor and myeloid-derived suppressor cells in the tissues of different organs [50]. The clinical consequence of this delayed and prolonged PAID was recently published in a monocentric retrospective study [51].

Lymphopenia observed at the beginning of sepsis was equally present at 28 days in survivors and non-survivors, with no difference between the two groups. As of day 4, the absolute mean lymphocytic count was higher in survivors than non-survivors and was independently associated with 28-day survival and increased development of secondary infections.

Multiple mechanisms can explain this adaptive immunity depression. The key point was the demonstration that apoptosis causes marked exhaustion of CD4, CD8 and B T cells in various organs in patients who die from septic shock, with no difference associated with age and type of pathogen. Sepsis-induced apoptosis can be activated either by the pathway triggered by the death receptor or by the metabolic pathway [43,52,53].

Immune cell activation is also regulated by metabolic pathways, so we can talk about “immune metabolism” [54]; in fact, mitochondrial ATP is obtained with these pathways, which is necessary to support immune function.

The energy needed is gained through two routes: first, via glycolysis and the tricarboxylic acid (TCA) cycle [55,56]; secondly, via the oxidation of fatty acids as a source for specific cell subgroups of T lymphocytes [54].

Therefore, metabolic changes in sepsis may be responsible for modifications in the immune system, but also may be a consequence of the disease. Unlike innate immunity cells, T cells can proliferate quickly and massively after activation, a process that uses Warburg’s metabolism (a high aerobic glycolysis rate). Activated T lymphocytes use oxidative phosphorylation and glycolysis, producing pyruvate, and they activate the pentose phosphate pathway, which can produce reactive oxygen species.

Memory T cells and Treg cells use oxidation of fatty acids to survive and support their functions [55].

When a naive T cell recognizes an antigen, it triggers a development program characterized by rapid growth, proliferation, and acquisition of specific functions and this requires metabolic reprogramming. This metabolic modification may influence the development and activity of T cell subgroups, as suggested for the proposed strict glycemic control [57,58]. A key question to be resolved is to understand if the PAID phenomenon described during sepsis is a “normal” adaptive response that follows the acute phase or if it represents immune system failure that must be treated.

### 3.3. Techniques That Are Useful for the Study of Circulating Cells in Sepsis and to Understand If Immune Cells Act as a “Biopsy Sample”

Various cellular and molecular biology techniques are currently available to study the immune status of patients with sepsis. For example, C-reactive protein (CRP) and procalcitonin (PCT) are often used in the clinical setting as parameters to manage infection and response to antibiotic therapy since they are indirect biomarker of infection, although their value is increased in other pathological conditions, such as trauma or major surgery [59,60].

ELISA has been used to evaluate plasma levels of molecules involved in inflammation and apoptosis, describing their association with mortality and disease scores.

Other molecules are involved in predicting the severity of sepsis, such as presepsin and serum angiopoietin (Ang)-2 [61,62].

The soluble CD40 ligand (sCD40L) shows pro-thrombotic and pro-inflammatory properties after binding to the CD40 cell receptor. Circulating levels of sCD40L are significantly higher in septic patients than in controls and non-survivors [63].

Huttunen et al. evaluated the prognostic value of apoptosis markers such as soluble Fas (sFas), Fas ligand (FasL) and the sFas/FasL ratio in patients with bacteremia, describing the direct association between these mediators and a high SOFA score [64].

**Figure 7:**
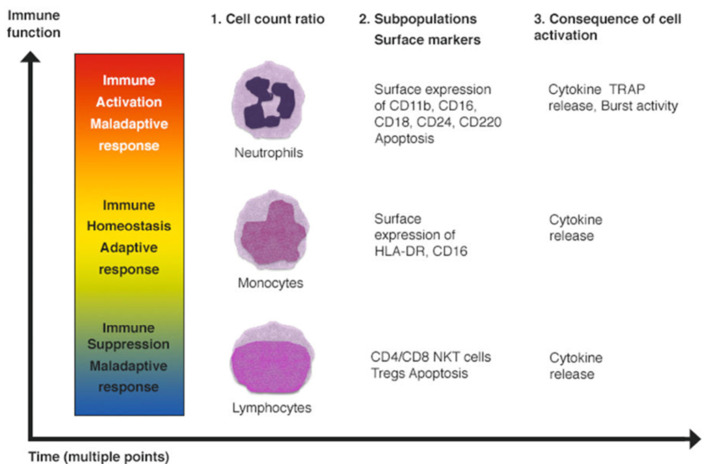
Immune cell activation, suppression and homeostasis. Fluorescence-activated cell sorting (FACS) can identify neutrophils, monocytes and lymphocytes through: (1) cell count ratios; (2) specific subpopulation surface markers; (3) biological changes.

Another technique used for the study of the immune status is fluorescence-activated cell separation (FACS). FACS enables the simultaneous determination of multiple antigens, highlighted with different fluorochromes, and can be used as a first tool to determine the quantity of specific immune cells (leukocyte typing). Specific staining of surface antigens can identify helper T cells, 1 or 2, and various lymphocyte subpopulations [65,66,67] (Figure 7).

FAC can be used to evaluate many parameters involved in sepsis—for example, integrin molecules whose expression is increased on leukocyte surfaces during inflammation, or the reduction in HLA-DR expression on monocytes, considered a predictor for mortality in severe sepsis [68,69].

In NK cells, the FACS analysis of NKG2D is used as a marker of cellular activation and the expression of CD107 to identify degranulation [70,71]. FACS is also able to distinguish between the expression of surface molecules and intracellular antigens: the intracellular levels of TLR2 and TLR4 in the NK cells of septic patients increased compared to those of healthy subjects [69]. Mariam Onsy F. Hanna et al. [72] demonstrated that the determination of CD64 expression on neutrophils by flow cytometry is useful for predicting sepsis in critically ill patients (OR = 1.04; *p* = 0.028). The specificity of neutrophil CD64 for sepsis was 91%, with an AUC of 0.66, at the optimal cut-off of 54 MFI. In contrast, neutrophil CD16 and monocytes CD64 and CD14 lost the capacity to predict sepsis in critically ill patients. Moreover, they demonstrated that the neutrophil CD64/monocyte CD64 ratio can predict sepsis, with an OR of 91.55, although it did not reach statistical significance (*p* = 0.075), probably due to the poor campion size. Neutrophil CD16 expression achieved significance for prediction of mortality risk measured by the APACHE II score among all patients (sepsis and no sepsis patients; *p* = 0.025) using linear regression. Multi-variate regression analysis with sepsis as the dependent variable and patient parameters including neutrophil CD64, CRP and SOFA as well as the sepsis score as independent predictors showed that the score was independently associated with sepsis and was significant as a predictor of sepsis (OR = 47.5, *p* = 0.003).

Additional strategies are used—for example the new frontier of “OMICS” technology (genomics, transcriptomics, proteomics and metabolomics) may improve new approaches as a sort of “molecular microscope” to develop new diagnostic tools [73]. Studies examining single-nucleotide polymorphisms (SNPs) in sepsis have generated mixed results. However, the TNF SNP functional gene rs1800629 was strongly associated with susceptibility to sepsis [74].

Microarray analysis of genes and quantitative RT-PCR are now being used to advantage in the study of genes implicated in inflammation to confirm gene modulation [75]; RT-PCR has also been used to examine the gene-level reduction in HLA-DR expression in monocytes, with promising results [76]. In fact, gene profiling of leukocytes in the blood is being considered in the relationship between encoding of gene expression and related protein levels during sepsis.

Furthermore, RT-PCR in used in the measurement of mitochondrial or cell-free DNA. Recent studies have highlighted the importance of epigenetics in immune dysfunction associated with sepsis, through DNA methylation and histone acetylation in inflammatory genes [77]. Among epigenetic mechanisms, microRNAs, small non-coding RNAs capable of modifying gene expression in target cells, are modulated in plasma during sepsis. MicroRNAs are analyzed and identified by RT-PCR; miR-15a, miR-16, miR-122, miR-133, miR-193, miR-223 and miR-483-5p are all increased in human sepsis and are associated with a higher mortality rate [78,79,80].

In the future, research is based on the study of exosomes/circulating extracellular micro vesicles (EVs); EVs are potential emerging biomarkers of diseases, because they seem to be involved in the transfer of material between cells (for example of proteins, receptors, bioactive lipids and genetic material as mRNA and microRNA). EVs can also be detected in plasma by FACS or with specific techniques such as Nanotrack analysis [81]. Circulating vesicles may also appear to be involved in the tissue damage of the myocardium and endothelium caused by sepsis [82,83].

In conclusion, the possibility of analyzing the genome and the continuous development of new technologies will broaden knowledge of the immune status in septic patients in the coming years. This will allow the development of new effective personalized molecular treatments for this disease.

### 3.4. Can Extracorporeal and Non-Blood Purification Therapies Alter Cell Phenotypes and/or Change the Function of Leukocytes?

Therapeutic modulation of the host’s immune response during sepsis has always been a challenge. In recent decades, several attempts have failed to demonstrate any benefit in terms of improving patient outcomes [84,85]. 

Currently, it appears that extracorporeal therapies are giving good results. Among these strategies, the whole panel of extracorporeal blood purification therapies appears to be among those with the best impact on the septic patient’s immune system: they manage to change the phenotype of immune cells and/or their function.

Several new molecules have recently shown very promising effects in this field and are also currently being tested. More traditional strategies such as high-volume hemofiltration and its variants (high-volume pulsed hemofiltration and cascade hemofiltration) have concentrated their action on the possibility of removing inflammatory mediators from the blood, a phenomenon that can subsequently lead to a change in the phenotype of leukocytes and in their function. In a pig model, Yekebas et al. reported that high-volume endotoxin hemofiltration in vitro may prevent the sepsis-induced hyporesponsiveness [86].

Among extracorporeal blood purification strategies, there is also hemoperfusion, based on the removal of target molecules from the blood by filtering. The filter using polymyxin-B affects endotoxins and has been shown to restore the expression of HLA-DR on monocytes and CD16 on granulocytes with a decrease in IL-10 levels [87].

Opinions on this technique are still divergent. In fact, Payen et al. have recently reported a negative opinion on this therapy [88]; instead, Kumagai et al. have pointed out another ability of this type of sorbent, which is that it is able to selectively remove activated neutrophils which express high levels of CD11b/CD64 and low levels of CXCR1/CXCR2. Removal of this cell has been associated with an ex vivo reduction in the ability of circulating cells to cause damage to single-layer endothelial tissue [89].

Srisawat N et al. [90] conducted a randomized controlled trial in patients with blood endotoxin activity assay levels treated with polymyxin-hemoperfusion and compared with a group who received a standard treatment. They enrolled 55 patients (PMX-HP vs. standard treatment) and an improvement in “leukocyte reprogramming” was observed. PMX-HP therapy enables a higher expression of mHLA-DR compared to in patients who received the standard treatment (*p* = 0.027). The PMX-HP treatment improves outcomes of sepsis/septic shock in patients, significantly reducing CD11b expression on neutrophils, and should be considered a potential treatment strategy. 

Hemoperfusion with Cytosorb technology can remove both activated leukocytes and cytokines from circulation [91,92]. Furthermore, the removal of cytokines and chemokines through this technique is likely to modify the local chemokine gradients between the infection site and the plasma, and thus lead to greater enrollment of leukocytes [93].

The hybrid blood purification technique called plasma filtration adsorption (CPFA) plays an important role as it adsorbs inflammatory mediators through a special resin that functions as a filter. In fact, Ronco et al. reported that CPFA may restore leukocyte responsiveness to LPS in a prospective crossover clinical trial in which patients with septic shock were enrolled [94].

We know that one of the most serious damages during sepsis occurs at renal tubular cells, caused by circulating inflammation mediators [95]. In septic patients with acute renal damage, the use of filters with high cut-off membranes is able to reduce the phagocytosis of polymorphonuclear neutrophils and restore the peripheral proliferation of mononuclear blood cells [96,97].

In a randomized, double-blind, placebo-controlled pilot study, Leentjens et al. reported that interferon-γ may attenuate the LPS-induced reduction in the TNF-a response and may increase the expression of HLA-DR on monocytes [98]. Meisel et al. have demonstrated in a randomized controlled trial that GM-CSF is also able to reverse monocyte inactivation (demonstrated by the increase in the expression of HLA-DR on the monocyte) and to restore induced pro-inflammatory monocytic cytokine production ex vivo from TLR-2/4. Interestingly, some positive clinical effects were also observed, such as a shorter time of mechanical ventilation and a shorter ICU stay in the GM-CSF group [99]. In vitro blockade of the PD-1/PD-L1 pathway can also have an impact on the phenotype of immune cells and on the function of leukocytes with a decrease in lymphocytic apoptosis and the restoration of the ability of immune effector cells to produce cytokines such as interferon γ and IL-2, which are essential for host immunity [100]. Finally, Venet et al. have shown that ex vivo treatment with recombinant human IL-7 can improve lymphocyte function, with an increase in the proliferation of CD4+ and CD8+ T cells, an increase in the production of interferon-γ by lymphocytes, an increase in the phosphorylation of the molecule key signaling called STAT5 (signal transducer and transcription activator 5) and an increase in the induction of B cell lymphoma 2 [101] (Table 3).

In conclusion, there are many promising techniques capable of acting on the immune system and on a patient’s immune response. It is important to underline that these potential strategies can have effects on the three different levels that we have discussed: the number of immune cells, the proportion of cellular subpopulations through the modification of surface markers expressed on leukocytes, and cellular expression and function.

### 3.5. COVID-19 and “Cytokine Storm”: The Role of Blood Purification

In December 2019, a series of unexplained pneumonia cases appeared in Wuhan. The new disease was defined as coronavirus disease-19 (COVID-19), an infectious pathology caused by the SARS-CoV-2 virus, by the World Health Organization (WHO). In most cases, SARS-CoV-2 presents with fever and mild respiratory symptoms [102,103] but 13.8–25.5% of patients may develop more serious manifestations due to increased lung damage with possible development of acute respiratory distress syndrome (ARDS); among these patients, approximately 5–6% require admission to intensive care units [104,105]. The latter patients were characterized by the presence of severe respiratory insufficiency requiring mechanical ventilation, or shock, or multi-organ failure syndrome. 

Multi-organ involvement was also found in patients with severe disease such as gastrointestinal [106], coagulation [107], and kidney [108]. On the other hand, according to the available literature, it seems that the percentage of AKI does not increase among patients with COVID-19. In a Chinese cohort of 1099 patients with COVID-19, 93.6% were hospitalized, 91.1% had pneumonia, 5.3% were admitted to the ICU, 3.4% had acute respiratory distress syndrome (ARDS) and only 0.5% had AKI. However, COVID-19 in combination with AKI resulted in higher mortality [109].

In the peripheral blood of patients with COVID-19, modifications of PMNs have been found. Lymphopenia was found in 83.2% of COVID-19-positive patients and the prognosis of the disease was directly related to a decrease in circulating lymphocytes [109].

Numerous studies confirmed the reduction in CD4 and CD8 T lymphocytes, but an increasing number of inflammation indices were observed at the same time, e.g., interleukin (IL)-6, tumor necrosis factor a (TNF-a), IL-2, monocytes chemokine-1 (MCP-1), and macrophage inflammatory protein 1a (MIP1A). 

This huge release of cytokines into the blood is defined as a ‘cytokine storm’. Cytokine release syndrome (CRS) [110,111,112] leads to MODS and ARDS [113,114]; this is also responsible for multi-organ dysfunction and sepsis [115,116].

Considering the knowledge acquired on the treatment of sepsis using blood purification, it was decided to apply the same treatment to septic COVID-19 patients.

The Chinese National Health Commission proposed blood purification therapies for COVID-19-positive patients with a strong immune response. Furthermore, studies conducted in patients with increased cytokine levels and imaging indicative of inflammatory status demonstrated the importance of early treatment with continuous renal replacement therapy (CRRT) and immunoadsorption [117]. Treatment with CRRT in patients with severe MERS demonstrated effectiveness [118]. 

On the one hand, CRRT therapy has not achieved unanimous results; on the other hand, blood purification therapies seem to be successful in severe COVID-19. Initially, blood purification studies were conducted on critical patients with unstable circulatory status, also advising their use in severe COVID-19 [119,120,121]. 

Further, the literature reminds us to regularly monitor the inflammatory status of severe COVID-19 patients, determine the clearance level of crucial inflammatory factors that have a life of a few minutes, and consider the combination of other dialysis modes in addition to conventional CRRT. Early application of blood purification therapies in severe COVID-19 patients may achieve better efficacy and realize therapeutic goals such as stabilizing hemodynamics and improving MODS.

Another group of researchers, Padala et al., reported on their experience of using the oXiris^®^ filter in the treatment of COVID-19 patients. They demonstrated that early initiation of CVVHDF with the oXiris^®^ filter with systemic heparin anticoagulation may result in a decline in inflammatory markers [122]. OXiris^®^ is a particular and innovative membrane, which has the ability to remove both endotoxins and cytokines; it also replaces renal function and has antithrombogenic properties. The oXiris^®^ membrane is therefore made of three different layers, and this unique design enables the combination of four properties in one device: renal support, cytokine removal, endotoxin removal, and local anticoagulant treatment [123].

## 4. Conclusions

The most important alteration observed in septic neutrophils is the activation of a survival program capable of resisting apoptotic death. In septic monocytes, a reduced characteristic expression of HLA-DR is observed, but the antimicrobial function of these cells does not seem to be significantly altered in sepsis. As regards adaptive immunity, sepsis-induced apoptosis leads to lymphopenia in patients with septic shock and this process involves all types of T cells (CD4, CD8 and Natural Killer), except for regulatory T cells, which retain their function. Several promising therapies that target the host’s immune response to sepsis are currently under evaluation. These potential treatments can influence the count of immune cells, the percentage of cell subtypes and their function. During the worldwide pandemic caused by SARS-CoV-2, it was useful to study the ‘cytokine storm’ in order to find the best treatment. In fact, an additional treatment using the oXiris^®^ filter with systemic heparin anticoagulation was proposed. This treatment can decrease the concentration of inflammatory markers that affect the severity of the disease.

Other clinical studies are now awaited to confirm the promising preliminary results obtained from these therapies.

## Figures and Tables

**Table 1:** Suspected Infection Variables and definition of sepsis and septic shock.

*GENERAL VARIABLES*	*CUT-OFF*
Fever	>38.3 °C
Hypotermia	<36 °C
Heart Rate	>90 min or more than two S.D. above the normal value for age
Tachypnea	>20 rr/min
Altered Mental Status	impairment
Significant Edema or positive fluid balance	>20 ml/kg over 24 h
Hyperglycemia	pGluc > 140 mg/dl in the absence of diabetes
***INFLAMMATORY VARIABLES***	***CUT-OFF***
Leukocytosis	WBC count > 12,000/µL
Leukopenia	WBC count < 12,000/µL
***DISEASE***	***DEFINITION (SEPSIS-3)***
Sepsis	Suspected/confirmed infection+≥2 criteria of SOFA
Septic Shock	Sepsis+ fluid refractory Hypotension_ - Lactate 2 mmol/l - Vasopressor for MAP ≥ 60 mmHg

**Table 2:** Sequential Organ Failure Assessment (SOFA) Criteria. Adrenergic agents administered for at least one hour (doses given are in μg/kg/minute). Norepi = norepinephrine; dpx = dopamine; dbx = dobutamine; epi = epinephrine. ** PaO2/FiO2 = arterial partial pressure of oxygen/fraction of inspired oxygen + MAP = mean arterial pressure # With respiratory support.

SOFA	0	1	2	3	4
RESPIRATION (P/F) **	≥400	<400	<300	<200 ^#^	<100 ^#^
**COAGULATION (plts)**	≥150	<150	<100	<50	20
**LIVER, BILIRUBIN (mg/dL)**	1.2	1.2–1.9	2.0–5.9	6–11.9	>12
**CARDIOVASULAR**	Map^+^ ≥ 70	Map^+^ < 70	any dose dpx or dbx	dpx > 5 or epi ≤ 0.1 or norepi ≤ 0.1	dpx > 15 or epi > 0.1 or norepi ≤ 0.1
**GLASGOW COMA SCORE**	15	13–14	10–12	6–9	<6
**CREATININA (mg/dL)**	<1.4	1.4–1.9	2.0–3.4	3.5–4.9	>5.0

**Table 3:** Graphical overview of the current available devices to perform blood purification in critically ill patients affected by septic shock.

Extracorporeal Blood Purification			
Convection Therapies	Adsorption Therapies	Combination Therapies	Other Therapies
Continuous Renal Replacement (CRRT)	Immobilized Polimixin B (PMX)	Coupled Plasma Filtration Adsorption (CPFA)	Plasma exchange
High-Volume Hemofiltration (HVHF)	Hemadsorption (e.g., CytoSorb)	Combined Filtration and Adsorption (e.g., oXiris)	Renal Assist Device (RAD)
High Cut-Off Membranes (HCO)			
